# Financial forecasts accuracy in Brazil’s social security system

**DOI:** 10.1371/journal.pone.0184353

**Published:** 2017-08-31

**Authors:** Carlos Patrick Alves da Silva, Claudio Alberto Castelo Branco Puty, Marcelino Silva da Silva, Solon Venâncio de Carvalho, Carlos Renato Lisboa Francês

**Affiliations:** 1 Laboratory of Social Technologies, Postgraduate Program in Electrical Engineering, Federal University of Pará, Belém, Pará, Brazil; 2 Faculty of Economics, Federal University of Pará, Belém, Pará, Brazil; 3 Associate Laboratory of Computation and Applied Mathematics, National Institute for Space Research, São José dos Campos, Brazil; Utah State University, UNITED STATES

## Abstract

Long-term social security statistical forecasts produced and disseminated by the Brazilian government aim to provide accurate results that would serve as background information for optimal policy decisions. These forecasts are being used as support for the government’s proposed pension reform that plans to radically change the Brazilian Constitution insofar as Social Security is concerned. However, the reliability of official results is uncertain since no systematic evaluation of these forecasts has ever been published by the Brazilian government or anyone else. This paper aims to present a study of the accuracy and methodology of the instruments used by the Brazilian government to carry out long-term actuarial forecasts. We base our research on an empirical and probabilistic analysis of the official models. Our empirical analysis shows that the long-term Social Security forecasts are systematically biased in the short term and have significant errors that render them meaningless in the long run. Moreover, the low level of transparency in the methods impaired the replication of results published by the Brazilian Government and the use of outdated data compromises forecast results. In the theoretical analysis, based on a mathematical modeling approach, we discuss the complexity and limitations of the macroeconomic forecast through the computation of confidence intervals. We demonstrate the problems related to error measurement inherent to any forecasting process. We then extend this exercise to the computation of confidence intervals for Social Security forecasts. This mathematical exercise raises questions about the degree of reliability of the Social Security forecasts.

## Introduction

The concern with a crisis in public pension systems is not new [1] and policymakers around the world are increasingly concerned with finding alternatives to fund Social Security. In Brazil as well, not differently, the recent debate on the future of the National Social Security system is being driven by the fear that future retirements pose a real financial risk for the solvency of the System as discussed in [2].

The ongoing demographic change in Brazil, marked by an increase in life expectancy at birth, reduced mortality rate, continuous and persistent reduction in the fertility rate and increased life expectancy of people at older ages, will lead to radical changes in the actuarial mechanism of Social Security. These changes are due to both increases in expenses (an increase in the number of inactive elderly people and a longer duration of the benefits received) and to the reduction in the number of taxpayers due to the shrinking economically active population over time. These factors imply additional pressure to the current Social Security system, suggesting the need to evaluate its adequacy to the new demographic reality.

The general Social Security model in Brazil is the so-called pay-as-you-go (PAYGO) system, a mandatory transfer of income from the employed labor force to the elderly portion of the population. In this case, a public agency (Social Security), acts as a buffer between the two parts of the population [3]. In the PAYGO pension plan, the benefits are directly tied to the contributions or taxes paid by individual participants. The right to receive a pension is essentially a political right, the terms of which are guaranteed by the government, although the government might subsequently alter the terms on which pensions are conceded. In this model, the transfer of goods and services from the workforce to the pensioners is very transparent [4].

The most important set of long-term statistical forecasts produced and disseminated by the Brazilian government are population growth forecasts and financial estimates of Brazil’s General Social Security System (RGPS). If we consider that these two sets of forecasts are strongly correlated, assumptions in both will have great impacts on expected Social Security results.

Social Security financial forecasts formally aim at providing accurate results that would serve as background information for (socially determined) optimal policy decisions regarding the Social Security System. In fact, the public debate about the probable future of Brazilian Social Security has been quite intense since the inception of RGPS in the first constitution after the military dictatorship (1964-1985) in 1988 and with the enactment of laws 8212 and 8213 in 1991 that established the details and regulatory framework of the System. The main evidence of the political concern with the future of Social Security—and its role in public budget—are the various reforms that have taken place in the years since 1988, whose objects were both the national pensions and the special systems, like the federal public servants’ pension system and the (multiple) state governments’ pension systems.

Pressure for Social Security reform tends to remain on the Brazilian political agenda for many reasons, particularly because of fiscal problems that have arisen from the economic recession that has led to frustration with government revenues. At the end of 2016, the Brazilian government proposed changes to various Social Security rules, including a revision of the minimum age for retirement.

Faced with such important questions for the lives of millions of Brazilians, one expects that the terms of the debate would be accessible to as many people as possible, particularly to decision makers in government and in the National Congress. The basic requirement for democratic debate in this case is to make publicly available the instruments used by the government to evaluate present and future conditions of Social Security, particularly the RGPS. Therefore, databases and demographic forecasting models used to forecast the sustainability of Social Security should be broadly understood.

Not by accident, Article 4, paragraph 2, item III of the complementary law no. 101 of 2000 (the so-called Fiscal Responsibility Law) established that the Budgetary Guidelines Law (LDO) must contain an annex of fiscal targets including an evaluation of the financial situation of the general and special Social Security regimes. We assume that the legislator’s intention was to ensure higher transparency and reliability in the decision-making process involved in both budget cycle and multi-annual planning.

Therefore, from the 2002 LDO on, we can find an annex of fiscal targets with forecasts of RGPS result (commonly in Annex IV, but in some LDOs it may be in Annex III). These forecasts are conducted by the Secretary of Social Security Policies (SPS) of the Ministry of Social Security (MPS)—recently absorbed by the Ministry of Finance—and have been used by successive governments for the debate at the National Congress on the financial sustainability of the RGPS.

However, a closer look at Annex IV of the LDO clearly shows, despite the relevance of the topic, a remarkably low level of transparency in the methods used for forecasting Social Security results. Firstly, the model described in Annex IV is not replicable because it is simply incomplete. There is not, on the other hand, any other official document describing it in its original form or a description of the changes that it allegedly suffered during the last eleven years of the existence of Annex IV. Also, year after year, there is no process of (self) evaluation of the efficiency of forecasts. A simple comparison of forecasts against the budget execution would be enough to show the appalling forecast errors. Each LDO presents new forecasts, without making any mention to corresponding forecast results published in the past budget guidelines. Finally, and even more significantly, no executive order at any level of federal government establishes formal parameters, databases and specific methods for forecasts. Official Social Security forecasts simply do not have any regulation.

As we will show in the following sections, the long-term Social Security forecasts are systematically biased in the short term and have significant errors that render them meaningless in the long run. Moreover, forecast models used in LDO have an obvious stochastic character and, despite the various references in the text to the limitations of the model, their results are always presented without even specifying the margin of forecast errors, as if they were simply deterministic results.

Furthermore, as there is no official evaluation of the forecast model’s effectiveness, the results published in Annex IV of LDO are informally reviewed, but the nature of such revision and the impact of the decisions guided by such models are unknown to the broader public. We obviously understand that actuarial forecasts can not be totally accurate. However, the long-term forecasts carried out by the Brazilian government serve as input to a set of relevant strategic decisions, and its limits have to be more clearly explained, and the use of more recent—and less pretentious—forecast techniques should be used to a more robust and reliable decision-making support system.

This paper aims to present a study of the accuracy and methodology of the instruments used by the Brazilian government to carry out the long-term actuarial forecasts of RGPS. We, therefore, base our research on an empirical and probabilistic analysis of the official models. Such a study is of the utmost importance since, as we have mentioned, such forecasts are being used as support for the government’s proposed pension reform, which plans to radically change the Brazilian Constitution insofar as Social Security is concerned.

We have divided this study into two parts. The first part performs an empirical analysis based on official data provided by the Brazilian government. We look at the forecast results presented in the LDO, show its forecast errors and demonstrate their low reliability. We also try to replicate the LDO results following the model described in the official document. Finally, we conduct an analysis of the data sets used in the forecasts, showing that the Brazilian government has systematically used outdated data, which has an adverse impact on results.

In the second part, we conduct a theoretical analysis based on a mathematical modeling approach. From a standard exercise with Brazilian Gross Domestic Product (GDP) series, we discuss the complexity and limitations of macroeconomic forecasts through the computation of confidence intervals. We demonstrate the problems related to error measurement inherent in any forecasting process. We then extend this exercise to the computation of confidence intervals for Social Security forecasts as it is well known that pension revenues are closely related to GDP behavior. This mathematical exercise raises questions about the degree of reliability of the LDO forecasts.

The remainder of the paper is organized as follows. In the first section, we present a brief description of the Brazilian General Social Security System. Then, we describe some related works on Social Security financial forecasts. Next, we perform a comparison of Social Security forecasts in each Annex IV of the LDOs from 2002 to 2015 with its corresponding published official statistics. In the following section, we describe the forecast model used by the Brazilian government and present an attempt to replicate the financial results of the 2012 LDO. We then analyze population and labor market data sets utilized in each LDO forecast, showing the impact of outdated statistics on Social Security results. Next, we present the aforementioned theoretical analysis of GPD and Social Security forecasts. In the last Section, we present conclusions and suggestions to improve the Brazilian Social Security forecast system.

## Social security financing

As described in the Brazilian Constitution and regulated by Law nº 8212/91, Social Security comprises the right to health, pension, and social assistance. In concrete terms, it involves the payment of pensions with the purpose of guaranteeing its beneficiaries with essential means of maintenance. The right to a pension may be due to incapacity, advanced age, time of service, involuntary unemployment, imprisonment of a family member or death benefits for those who are economically dependent. There are a different set of rules for urban and rural workers as well as men and women.

The largest Social Security system in Brazil is the aforementioned General Social Security System (RGPS), the federal system for employees under the rules established by the so-called Consolidation of Labor Laws (CLT in Portuguese), mostly workers within the private sector but also workers in some state owned enterprises and agencies at the local or national level. Local and state governments also have their own Social Security systems for their public servants as well as federal public servants and military personnel. RGPS is by far the largest pension system in Brazil and monthly pays more than thirty million beneficiaries around the entire country.

**Fig 1:**
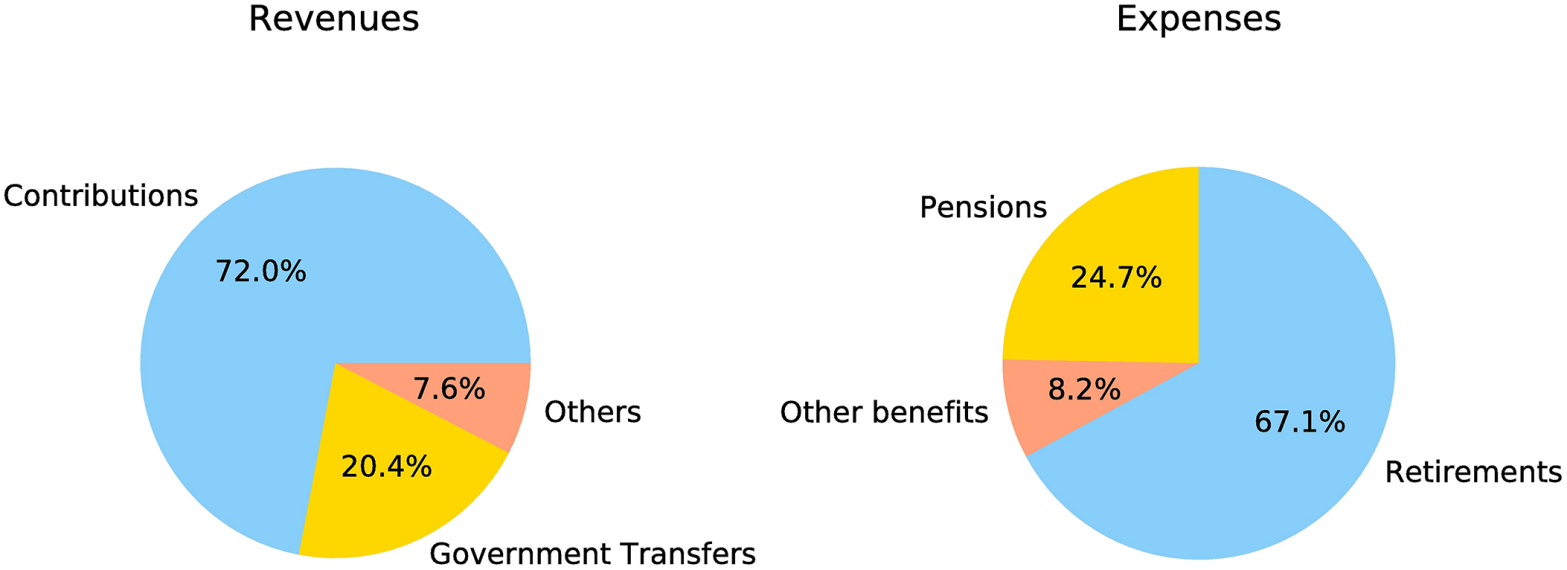
Distribution of the main revenues and expenses of the general system of social security in Brazil—2015.

RGPS is a pay-as-you-go system of pension and it is funded by a combination of government transfers and payroll contributions by employers and employees. Hence, it is a tripartite system based on an intergenerational simple distribution. Accordingly, article 10 of Law No. 8212/91 establishes that Social Security will be financed by the entire society, directly and indirectly in the form of social security contributions. Table 1 describes the primary sources of Social Security revenues and expenses as described in the Social Security Statistical Yearbook (AEPS) [5]. Also, Fig 1 shows the distribution of each source of revenues and expenses. It is worth mentioning that each source of income and expense has several subtypes according to the type of employment. For a detailed description of all sources of revenues and expenses, one can check Tables 41.8, and 42.5 in [5].

**Table 1:** Sources of revenue and main expenses of the general system of social security in Brazil—2015.

Revenues	Expenses
Social Security Contributions	Retirements
Government/Union Transfers	Pensions
Property Revenue	Other benefits
Capital Income	
Intra-budgetary Revenue	
Other Current Income	

In the thirteen years until 2015, Brazil underwent a robust process of social inclusion with the lowest unemployment rates in history and the creation of innovative social policies that have allowed millions of Brazilians to access Social Security. On the other hand, this process has made the rate of growth of Social Security expenditures surpass the rate of growth of revenues that in combination with an increasing elder population has raised some concerns about the necessity of a reform in the national public pension’s system. As the devil lives in the details, the calibration of any proposal of social security is crucial for social welfare and this is why we believe that efficient—or at least transparent—financial forecasting is crucial for the proper calibration of any proposal of reform.

## Related works

Concerns with the soundness and transparency of social security forecasts in the USA are present in the works of [6]. The authors offer an evaluation of the Social Security Administration demographic and financial forecasts used to assess the long-term solvency of the Social Security Trust Funds in the USA. A comparison of Social Security forecasts with the observed data shows that forecasting errors—as evaluated by how accurate the forecasts turned out to be—were approximately unbiased until 2000 and then became systematically biased and increasingly so over time. Also, most of the forecasting errors since 2000 are in the same direction, consistently misleading users of the forecasts to conclude that the Social Security Trust Funds are in better financial shape than turns out to be the case. Lastly, they show that the Social Security Administration’s informal uncertainty intervals appear to have become increasingly inaccurate since 2000.

In [7], the same authors show that the US Social Security Administration uses old statistical forecasting methods and makes public insufficient replication information. Their findings come from a large number of interviews with participants at every level of the forecasting and policy processes. A detailed evaluation of the methodology used to calculate mortality rates is described, showing several problems and the impact of the use of these rates on revenues and expenditures. They also suggest some manipulation due to political pressure. Finally, they make several proposals to solve the problems reported.

In [8] is presented a study that seeks to quantify, by using a long-term forecast model, the impact of demographics on the expenses of Social Security as a proportion of GDP for Brazil. Despite the great relevance of the results and the adequacy of the forecast model for the Brazilian case, the model used is quite simplistic. The model is useful only to illustrate the potential effects of aging on Social Security spending. It is inappropriate to forecast policy proposals concerning Social Security. Besides, there is no evaluation of forecast errors and confidence intervals.

In 2007, the Brazilian government promoted the National Forum on Social Security. One of the main products of this event was the document [9]. This document seeks to defend the rationality of parameters and structural elements of the long-term forecast model used by SPS/MPS while seeking to incorporate some of the suggestions made during the Forum. The document also mentions the use of two forecast models. One for the short term, i.e. for the current year and the subsequent three years, and another for the long term, starting from the fifth year following the current year. The short-term model is used to guide the preparation of the annual budget proposals, the Pluriannual Plans (PPAs) and to track revenues and expenditures in the current year. The long term model is used to simulate the impact of different proposals of structural reforms on Social Security financial balances. The document only describes some aspects of the long-term model without presenting it in detail. The authors refer to Annex III.5 of the 2008 LDO, where the model is explained in greater detail.

It is worth mentioning that for our study, most of the documents, papers, and databases made available by the Brazilian government are, as expected, in the Portuguese language.

## Analysis of the social security forecasts

As an initial part of this study, we have made a comparison between Social Security expenditure forecasts as presented in LDOs and the observed data on executed expenditures, available at InfoLogo, the official Social Security database [10]. Each LDO has forecasts of at least 20 years and the period analyzed was from 2002 to 2015. LDOs are produced one year before the corresponding year, i.e., the 2016 LDO was presented in Congress in April 2015. Also, as LDO is written one year before the corresponding year, the data used has a 2 years lag. Thus, the 2016 LDO is produced in 2015 using 2014 data.

**Fig 2:**
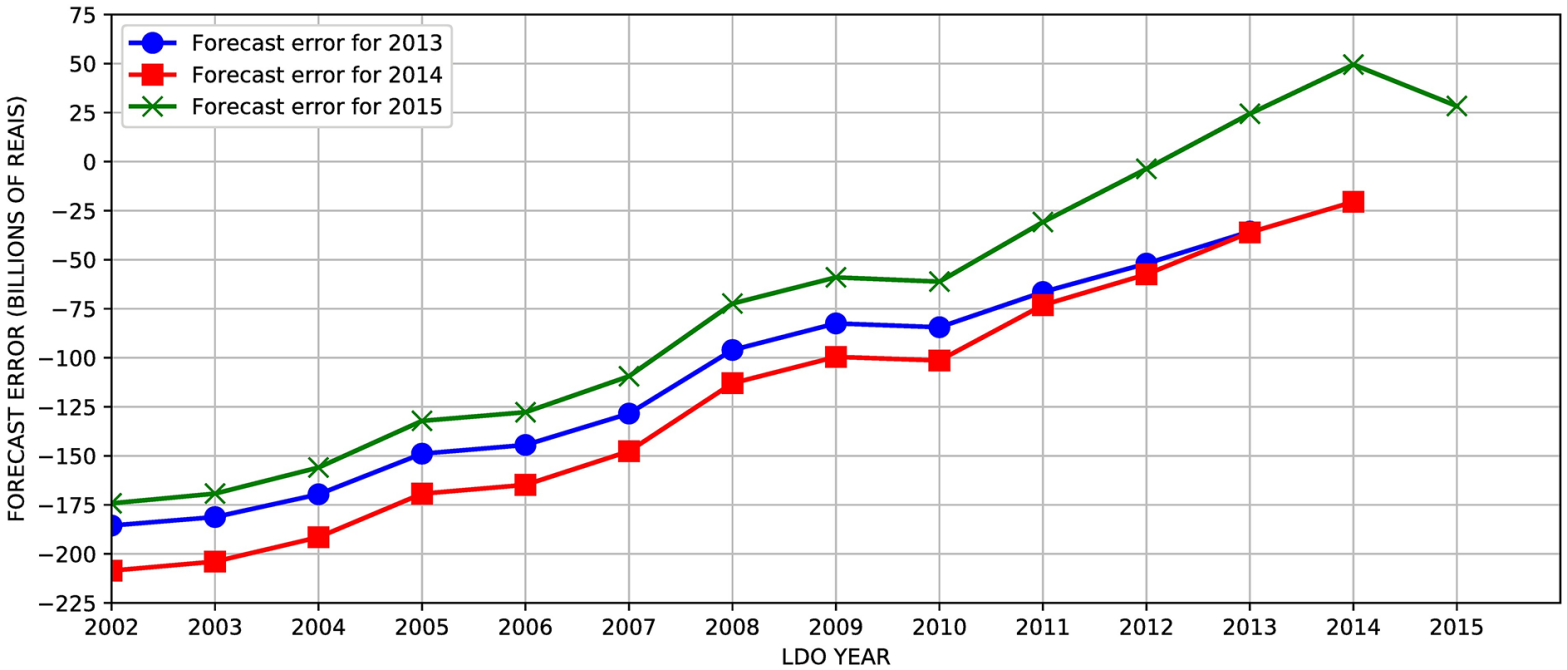
Forecast errors for revenues in each LDO for 2013, 2014 and 2015. Each curve shows the differences between the actual revenue value for a particular year (2013-2015) and the estimated revenue for these three years in each LDO from 2002 to 2015.

**Fig 3:**
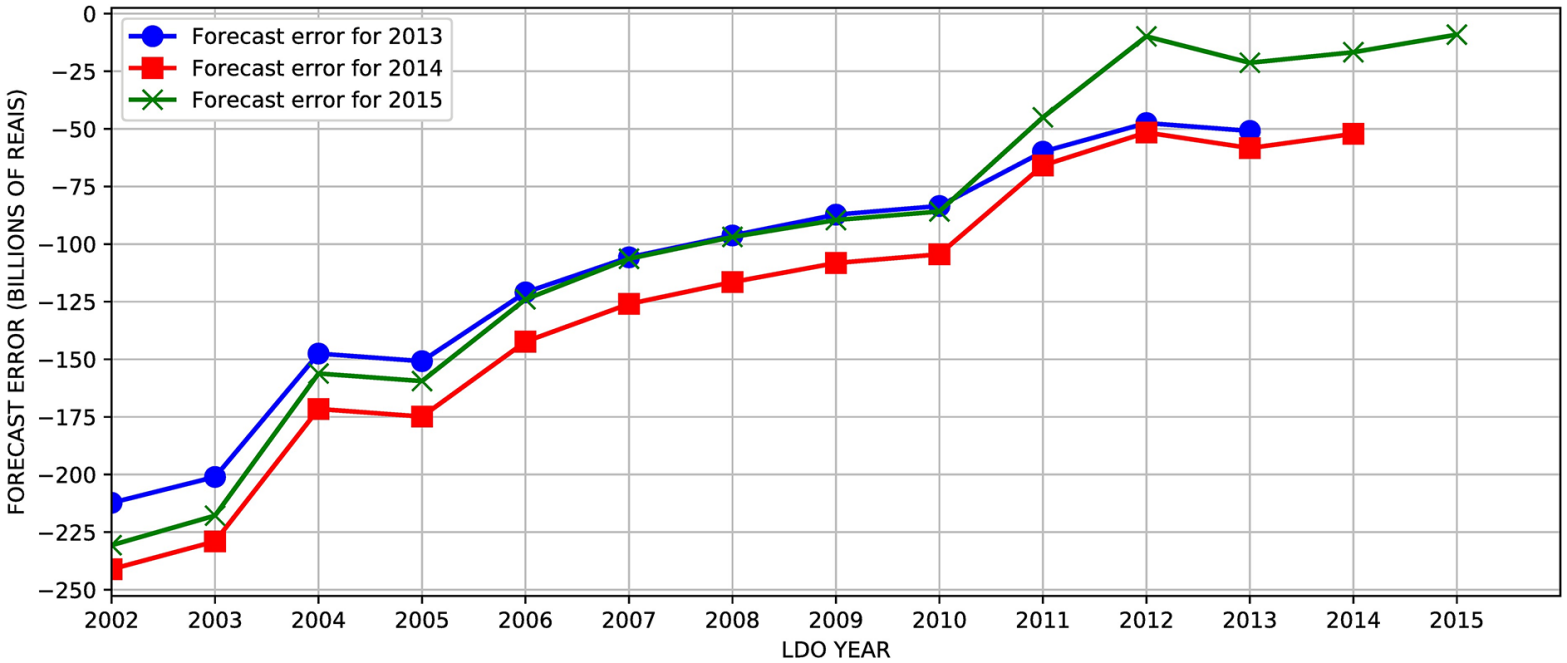
Forecast errors for expenses in each LDO for 2013, 2014 and 2015. Each curve shows the differences between the actual expense value for a particular year (2013-2015) and the estimated expenses for these three years in each LDO from 2002 to 2015.

For our comparisons between the forecasted results and the observed, we have chosen the three years with the highest number of mentions in the different LDOs since 2002, when the Social Security forecast reports began being officially reported. Figs 2 and 3 present the differences between the forecast value and the realized (observed) data—what we will refer to as forecast errors for revenue and expenditure for the years 2013 to 2015. The curve for the year 2014, for example, shows the differences between the multiple forecasts for each LDO between 2002 and 2015 and the realized value in each of the three years under observation (2013, 2014 and 2015).

The first aspect of revenue forecasts is the substantial error, in this case, of underestimation. Note that the error is higher as LDO moves away from the forecast date. An exception was the forecast revenue from the year 2013, explained by the sudden and sharp decline in economic activity observed in Brazil from 2014. In Fig 3, we observe that expenditure forecasts also show a pattern of systematic error, similar to what was observed for revenues, where the error is higher as the forecast point distances from its original year.

**Fig 4:**
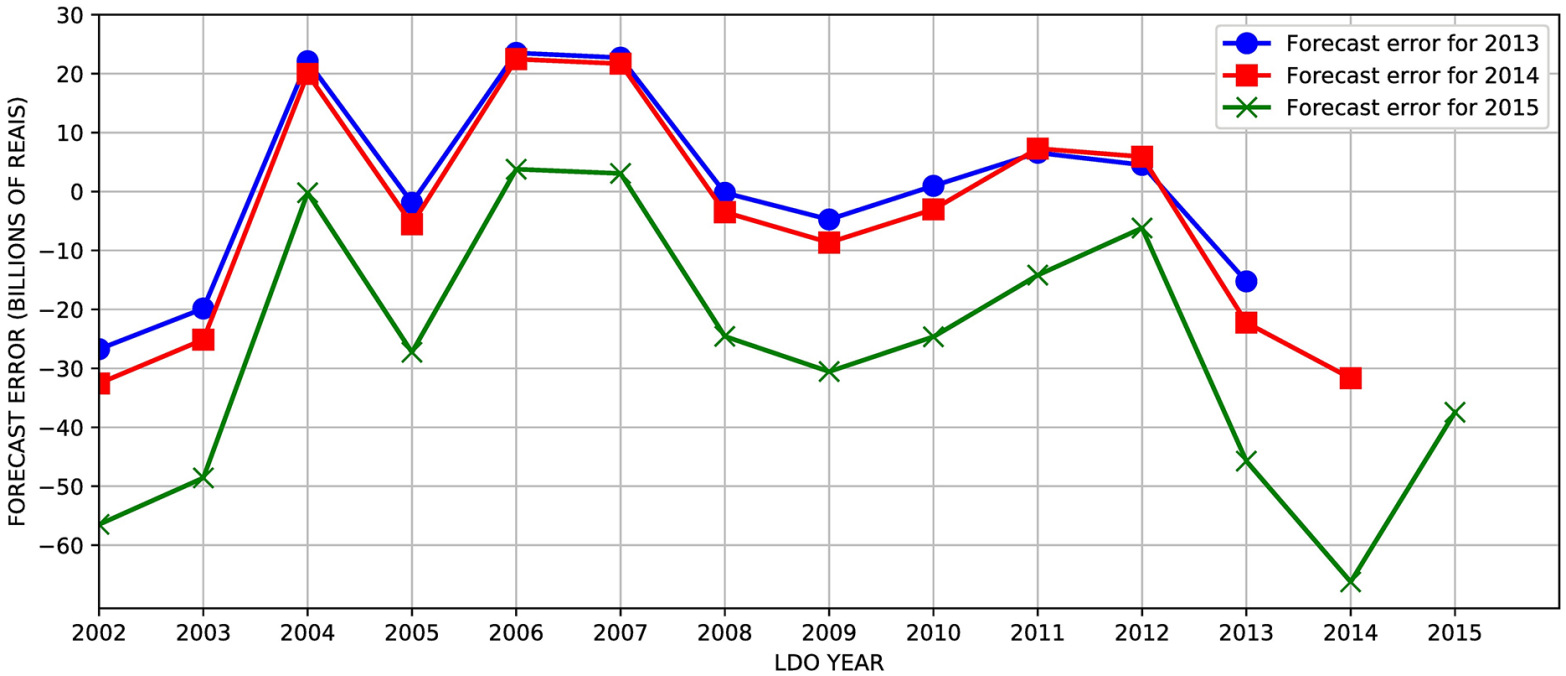
Forecast errors for financial results in each LDO for 2013, 2014 and 2015. Each curve shows the differences between the actual financial results for a particular year (2013-2015) and the estimated financial results for these three years in each LDO from 2002 to 2015.

The long-term solvency of Social Security is the difference between revenues and expenditures. Thus, we may have a situation of surplus or deficit of pension accounts. Fig 4 shows a bias of deficit overestimation until 2013, and from 2014, deficit underestimation. It is important to remember that we are dealing with forecast errors and the results shown in Fig 4 do not show the true financial result of the Social Security. Hence, an error of overestimation of the deficit does not mean that there was no deficit and vice versa.

A common feature of the three sets of forecasts presented, as one might expect, is the relation between forecast error size and the distance from the base year. In fact, an important source of distortion in forecasts comes from model contamination from short-term economic phenomena, which leads to a replication of prevailing economic conditions in the base year into the future. A forecast for a year of higher economic growth tends to replicate this effect for the future series and conversely in years of a stagnant economy. Hence, when we look at the forecasts for the year 2060 as presented in the latest LDOs, we realize they have little statistical validity since we perceive an explosion of the forecast error as we move away from the base year—and yet, no uncertainty intervals are reported by the government. The purpose of uncertainty estimates is to protect oneself from expressing overconfident conclusions from the results, and if estimates are consistently falling outside those uncertainty intervals, then revision and improvement in the forecasting process should follow [7]. One intuitive way to assess forecast errors would be breaking it down into demographic, economic (labor market) and Social Security variables. The difficulty here is the lack of records of the statistical series that were the basis for the LDO forecasts. We have the forecast value of revenue and expense, but we do not have their respective subcomponents.

### Results replication

At present, SPS at the Ministry of Finance is responsible for the forecasts depicted in Annex IV of LDO. However, as we have mentioned, SPS does not share sufficient information on forecast procedures and, as a result, no fully independent quantitative evaluation of results presented in LDO has been made possible. The possibility of replication and data sharing is a widely understood and accepted best practice throughout the scientific community [7].

In order to evaluate the results and find the source of errors presented in the previous section, we have extensively tried to reproduce the LDO results. Initially, we examined the Annexes of all LDOs from 2002 to 2017 and identified that the presented equations are precisely the same in all documents. However, from the 2009 LDO on, an updated version of the forecast model is mentioned, which, for example, purportedly adopted a new concept of participation rate and discarded the direct usage of the unemployment rate. Notwithstanding this fact, the equations specified have not changed at all and are still the same in all LDOs—and the unemployment rate is still used as a variable. Authors in [9] describe a set of improvements in the forecast model, like the inclusion of the employment formalization rate and the 2014 LDO mentions a second update of the forecast model. But again, no changes are performed in the model equations. Given these facts, it is not clear which model is used in the forecasts, greatly impairing independent results’ replication.

In an attempt to replicate the forecasts, we have chosen the 2012 LDO as a reference and a set of Social Security data obtained from DATAPREV, the Social Security Information and Technology Company.

#### Social security forecast model

The mathematical models used in forecasts of Social Security expenditures and revenues are described in Annex III, Fiscal Targets of 2012 LDO [11].

All variables in the equations have four or five indexes. The indexing parameters follow the definitions and domain sets described in Table 2.

**Table 2:** Indexing parameters.

Variable	Meaning	Values
a	age	0, 1, …, 80
y	year	2012, 2013, …, 2031
g	gender	Male or female
o	operation zone	Urban or rural
k	kind of benefit	Retirements due to age, disability and contribution time, health insurance and pensions

To calculate the expenses, firstly, one must calculate the number of current benefits, here referred to as stock. The stock is calculated by the method of flows, where the granting and cessation of benefits are estimated, and then the stock of benefits in a year is determined. The benefits grant flows are calculated as:

$$\begin{array}{c} {\text{In}}_{\text{b}}\left(\text{a},\text{y},\text{g},\text{o},\text{k}\right)=P\left(a,y,g,o\right)*P_{in}\left(a,y,g,o,k\right), \end{array}$$ (1)

where *In*_*b*_ is the inflow in the benefits of type *k* with age *a* in year *y* of gender *g*, operation zone *o*, *P* is the population and *P*_*in*_ is the probability of entry into benefit. The values of *P*_*in*_ are constant for all years and depend on the number of benefits granted and the population as presented in Eq (2).

$$\begin{array}{c} {\text{P}}_{\text{in}}\left(\text{a},\text{g},\text{o},\text{k}\right)=\frac{benef_{granted}\left(a,g,o,k\right)}{P(a,g,o,k)}, \end{array}$$ (2)

In turn, the stock of benefits is given by Eq (3):

$$\begin{array}{c} {\text{S}}_{\text{b}}\left(\text{a},\text{y},\text{g},\text{o},\text{k}\right)=S_{b}\left(a-1,y-1,g,o,k\right)*P_{surv}\left(a,y,g,o\right)+In_{b}\left(a,y,g,o,k\right), \end{array}$$ (3)

where *S*_*b*_ represents the stock of benefits of type *k*, *S*_*b*_(*a* − 1, *y* − 1, *g*, *o*, *k*) is the stock of the previous year, *P*_*surv*_ is the probability of an individual surviving from age *a* − 1 in year *y* − 1 to age *a* in year *y* and *In*_*b*_ are the entries (new beneficiaries). The probability of surviving *P*_*surv*_ is computed as depicted in Eq (4):

$$\begin{array}{c} {\text{P}}_{\text{surv}}\left(\text{a},\text{y},\text{g}\right)=\frac{P(a+1,y+1,g)}{P(a,y,g)} \end{array}$$ (4)

Then, the total stock of benefits in a year *y* is given by the sum of all benefits, for all ages, genders, and operation zones as described in Eq (5).

$$\begin{array}{c} \sum_{\text{a}}\sum_{\text{g}}\sum_{\text{o}}\sum_{\text{k}}{\text{S}}_{\text{b}}\left(\text{a},\text{y},\text{g},\text{o},\text{k}\right) \end{array}$$ (5)

Spending on benefits is calculated from the stock and the average value of the benefits, as shown in Eq (6).

$$\begin{array}{ccc} \text{E(a,y,g,o,k)} & = & E\left(a-1,y-1,g,o,k\right)*P_{surv}\left(a,y,g,o\right)*A_{b}\left(a,y,g,o,k\right)\\ & & +In_{b}\left(a,y,g,o,k\right)*Ain_{b}\left(a,y,g,o,k\right), \end{array}$$ (6)

where *E* is the expense with benefits, *E*(*a* − 1, *y* − 1, *g*, *o*, *k*) are the expenses in the previous year, *P*_*surv*_ is the probability of surviving, *A*_*b*_ is the annual average value of the benefit paid and *Ain*_*b*_ is the annual average value of the benefit paid for the input flow. The difference between *A*_*b*_ and *Ain*_*b*_ is that *A*_*b*_ takes into account benefits already granted that can not have their values reduced (only readjusted). However, *Ain*_*b*_ considers the amount paid to new beneficiaries, which may suffer variations due to changes in social security rules.

To calculate the revenue, one must estimate the number of taxpayers, according to Eq (7).

$$\begin{array}{ccc} \sum_{\text{a}}\sum_{\text{g}}\sum_{\text{o}}\text{C}\left(\text{a},\text{y},\text{g},\text{o}\right) & = & \sum_{a}\sum_{g}\sum_{o}P\left(a,y,g,o\right)*Part\left(a,y,g,o\right)\\ & & *[1-U(a,y,g,o)]*d(a,y,g,o) \end{array}$$ (7)

where *C* is the taxpayer stock, *P* is the population, *Part* is the labor force participation rate, *U* is the unemployment rate, and *d* is the contribution density. Contribution density represents the proportion of months that an employee contributes annually, i.e., a value of 1 means that an employee contributed every month in a year. Once the number of taxpayers is estimated, the value of revenues (*R*) in a year *y* is calculated as Eq (8).

$$\begin{array}{c} {\text{R}}_{\text{y}}=\sum_{a}\sum_{g}\sum_{o}C\left(a,y,g,o\right)*\left[r_{1}*Min\left(C_{e},S_{a}\left(a,y,g,o\right)\right)+r_{2}*S_{a}\left(a,y,g,o\right)\right)] \end{array}$$ (8)

where *r*_1_ is the Social Security contribution rate paid by the employee, *r*_2_ is the Social Security contribution rate paid by the company, *C*_*e*_ is the contribution ceiling (the maximum value used to calculate the contribution) and *S*_*a*_ is the employee salary. The *Min* in the equation ensures that the value used to calculate the employee contribution will not be greater than the ceiling (*C*_*e*_).

Here we have to explain an important point about pension income. As previously described, revenues from Social Security have several sources, but in the model presented, only direct contributions from employees and companies are considered (Eq (8)). Examining revenues from 2011 to 2015, we have observed that the sum of the contribution of employees and companies correspond, on average, to 55.7% of the total Social Security revenue, as described in Table 3. Thus, the model presented includes approximately half of Social Security revenues.

**Table 3:** Proportion of revenue from employees and companies’ contributions in relation to total revenue.

Year	CEC	TCR AESP	(%)
2011	143.3	242.2	59.1
2012	156.4	268.8	58.1
2013	159.5	292.6	54.5
2014	167.4	312.7	53.5
2015	170.3	319.6	53.2

CEC is the acronym for contribution from employees and companies, and TCR AESP is the total contribution revenue from AEPS, both in billions of reais.

#### Replication of social security forecasts

As mentioned above, we have chosen the 2012 LDO for forecast replication. The data sets and parameters used in the replication attempt are described in Table 4.

**Table 4:** Data sets and parameters used in the replication attempt of the results of the 2012 LDO.

Forecasted period	2012-2031
Population data	2008 IBGE forecasts
Labor market data	Table 5.1 in Annex IV of the 2012 LDO (Annex 4)
Stock data	DATAPREV data of 2010
Expenses and revenues data	DATAPREV data of 2010
Benefit Entry Probabilities	Based on the relative frequency of 2010 events
contribution density	1
employee contribution rate	0.08, 0.09 or 0.011 depending on the salary
employer contribution rate	0.2
Productivity growth per year	1.6%

Several variables described in the forecast model do not have their values or equations presented in the LDO, as, for example, the contribution density and probabilities of entry and survival. The probabilities of entry were calculated based on the flow of granted benefits in the recent years, and the probabilities of survival were calculated based on population data provided by Brazilian Institute of Geography and Statistics (IBGE) as previously described in Eqs (2) and (4), respectively.

As previously described, LDO mentions a short-term model that is used to forecast the first five years (2011 to 2015), and then a long-term model that is used for the remaining years. In [9], some differences between the models are described. The short term model has fewer variables and uses the forecast of some macroeconomic parameters provided by yet another branch of government—the Secretary of Economic Policy at the Ministry of Finance. The series for executed Social Security revenues and expenses are provided by the cash flow database of the National Institute of Social Security (INSS). Demographic parameters are not used in this model since, in the short term, demographic variations allegedly have a minimal impact on Social Security. In LDO, there is no description of the short-term model and no other document describing it has been provided by the government. Therefore, all the results presented here are based only on the long-term model. The long-term model is supposedly more complex and considers a much larger set of variables, including population forecasts. By using the demographic forecast, the long-term model incorporates the expected change in the age structure of the population.

**Fig 5:**
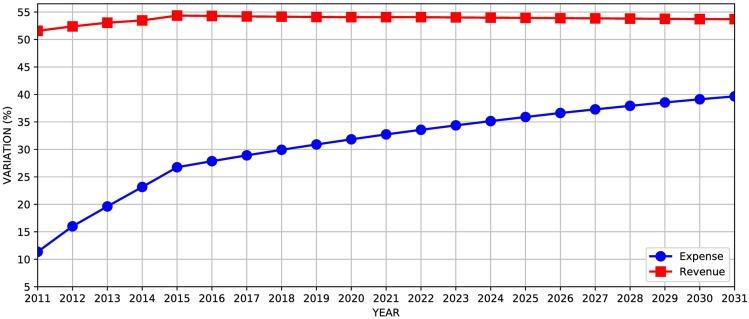
Variations (in %) between our results and LDO forecasts.

Once the model has been described, we have implemented and carried out forecasts of revenues and expenses of Social Security. Fig 5 show the variations (in %) between our results and LDO forecasts.

By following the equations presented in 2012 LDO we have tried to replicate the published series for forecasted expenses and revenues. In what concerns expenses, the variation in the first year is 11% and increases over the years, reaching 39% in 2031. There are several possible reasons for theses differences between our results and the official results. Firstly, as we have mentioned, there is no information about the short-term model. LDO mentions that the short-term results are provided by the Secretary of Economic Policy at the Ministry of Finance, following a so-called parameter grid released on April 8th, 2011. However, a closer examination of the document reveals no particularly useful information that could be used for modeling improvement. Secondly, there is a clear absence of complete data sets used in the forecasts. All necessary data should be described or, at least, referenced, but unfortunately, it is not available for independent exercises.

From the Fig 5, we observed an average variation of 53.7% in revenues, with small shifts. As the LDO does not describe any other source of revenue, and from Table 3, we have seen that the contribution of employees and companies corresponds to approximately half of the income from Social Security contributions, and the variation is almost constant throughout the forecast, we believe that some adjustment is applied to the forecast of each year to account for other incomes.

The failure in replication attempt reinforces the lack of transparency in the methods used by the Brazilian government to forecast revenue and expenses of Social Security. In future financial reports, the government should include a detailed description of methods, data used, a discussion of what forecasting mistakes were made in previous reports, what was learned from the mistakes, and what actions might be needed to improve forecasts. The above results were calculated using the MATLAB software, and all developed code and databases are available in [12].

### Analysis of data sets used

To carry out Social Security forecasts, two sets of data are essential: population forecasts and labor market statistics [13, 14]. Population forecasts are conducted by IBGE, the institute responsible for official population estimates. Currently, IBGE has four population projections, identified by the years in which they occurred: 2000, 2004, 2008 and 2013. Labor market datasets are provided by the National Household Sample Survey (PNAD), conducted annually by IBGE.

To ensure reliability in Social Security forecasts, it is crucial that an annual update is performed in the data sets used. Therefore, we expected that the most recent data available from one branch of national government would be applied in the forecasts. However, as described in Table 5, the population (IBGE) and the labor market (PNAD) data used in the forecasts take years to be updated. For example, data from PNAD of 2005 was used for LDOs from 2009 to 2013, but at no time in Annex IV of LDO was the use of lagged data justified.

**Table 5:** Year of data sets used in each forecast.

LDO Year	2008	2009	2010	2011	2012	2013	2014	2015	2016
IBGE data set	2004	2004	2008	2008	2008	2008	2008	2013	2013
PNAD data set	2000	2005	2005	2005	2005	2005	2009	2009	2009

**Fig 6:**
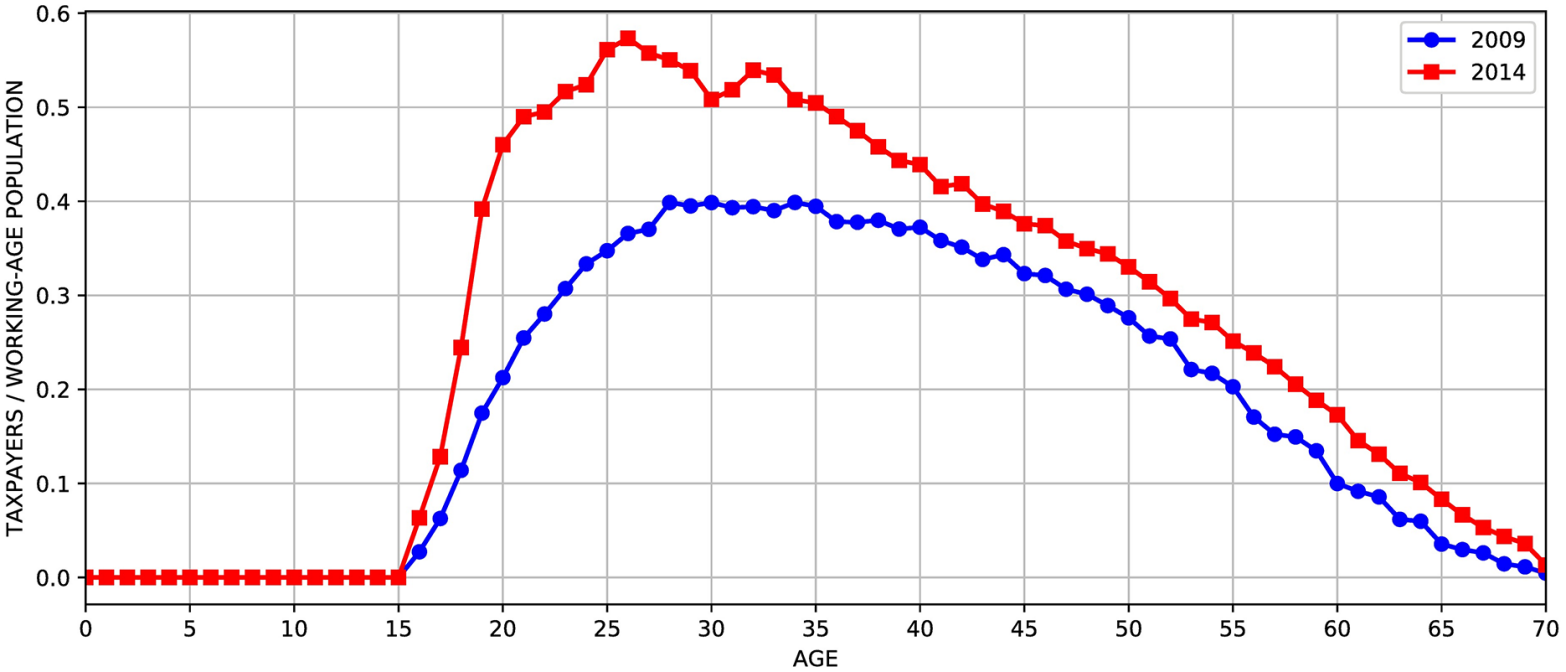
Labor market structure in 2009 and 2014 from PNAD of respective years. Each point in the graph represents the ratio of the number of taxpayers by the working-age population for each age.

As an example, Fig 6 shows the relationship between the total number of Social Security taxpayers and the working age population (16 to 64 years). We observe a clear right-upward shift of the curve, demonstrating a significant change in the labor market in a period in which government forecasts consider them constant. Variables such as participation rate and average salary have undergone significant changes but these movements are simply ignored in the forecast models.

A simple way to analyze the impact of labor market data can be made as follows. The number of employees can be calculated as:

$$\begin{array}{c} \text{Pop}\left(\text{u},\text{r}\right)=f\left(Pop_{total},Urb_{rate}\right) \end{array}$$ (9)

$$\begin{array}{c} \text{Emp(u,r)}=f(pop(u,r),Part,U) \end{array}$$ (10)

From Eq (9), the urban (*u*) and rural (*r*) population is calculated from the total population (*Pop*_*total*_) and urbanization rate (*Urb*_*rate*_). Then, from Eq (10), the number of employees (urban and rural, men and women) is a function of population, the participation rate in the labor force and the unemployment rate. So, the total amount of revenue is a function of employees, average wages and contribution rate as described in Eq (11).

$$\begin{array}{c} \text{Rev(u,r)}=f(Emp\left(u,r\right),Wages_{avg},Contrib_{rate}) \end{array}$$ (11)

As described in Table 5, the last four versions (from 2014) use fixed values of urbanization rates, labor force participation rate and unemployment, calculated from 2009 PNAD. In practice, the set of equations collapses, which makes the Eq (11) a simple function of the total population changes, independent of the labor market dynamics in the referred years. This makes, for example, the correlation between total revenues and the total population very high (0.8) from 2010.

**Fig 7:**
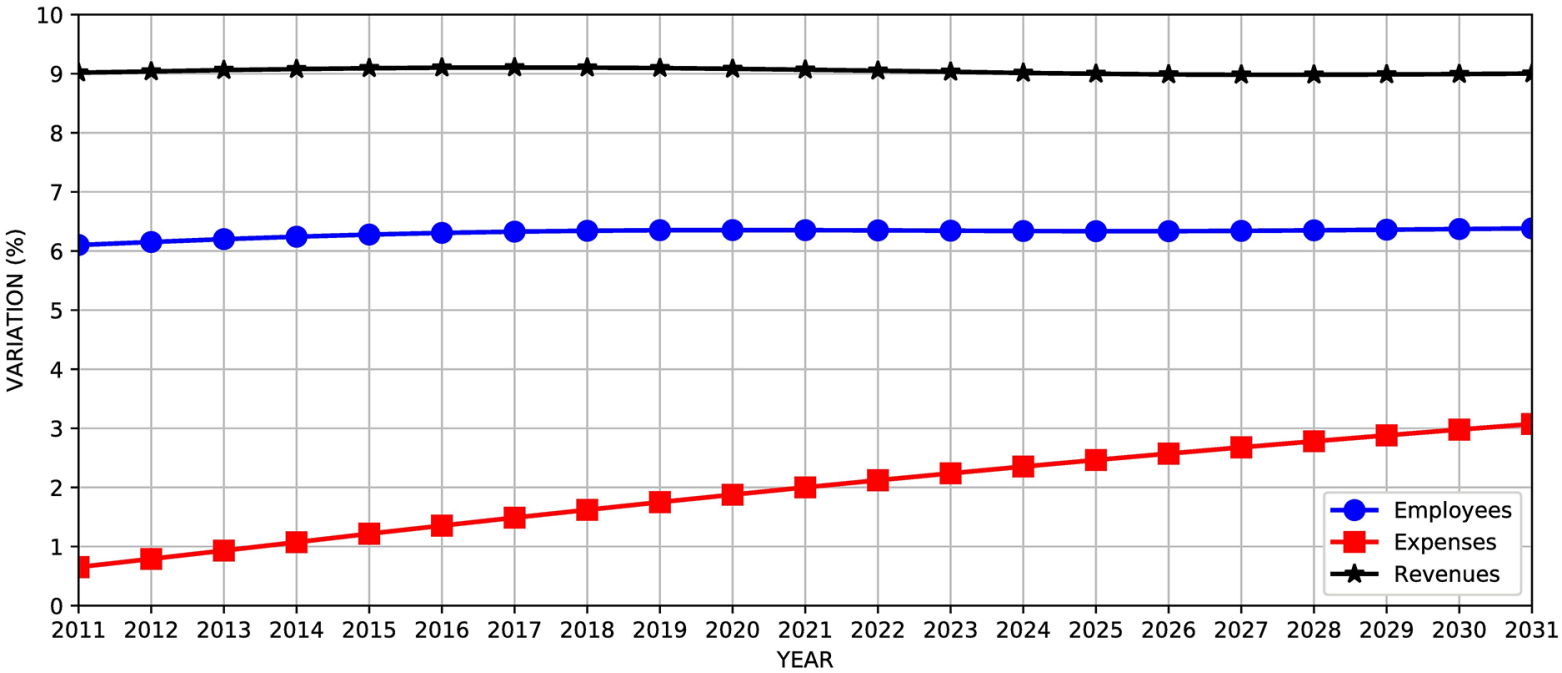
Variation in forecast results for revenues, expenses, and the number of employees when updating of labor market data from 2005 to 2009.

The use of outdated labor market data has great influence on results [14] and, in our opinion, compromises reliability. In another exercise, we revisited the forecasts presented in the previous section by changing the 2005 labor force participation data (used in the 2012 LDO) by 2009 (taken from the 2014 LDO). As shown in Fig 7, there is an average increase of 9% in revenue, 2% in expenditure and 6% in the number of employees.

## Dispersion calculation for GDP and social security forecasts

As we have seen, official forecasts are explicitly probabilistic but no reference to error margins are ever made in government documents. Even worse, forecast results are presented with a presumption of certainty with serious social and political consequences as they have embedded confidence intervals that are unknown to the audience. What would be the confidence intervals for the predicted series published in the budget guidelines? A simple exercise can be made starting from the GDP series. We have built a GDP forecast model from the information presented in Table 6, that corresponds to the same period of time for which pension data is available.

**Table 6:** GDP and population of Brazil with their respective annual growth rates.

Year	GDP	Population	Annual growth rates	
			GDP	Population
2000	3.917	173.447	0.1389896	0.01410940
2001	3.971	175.894	0.3053462	0.01360694
2002	4.092	178.288	0.1140829	0.01312190
2003	4.139	180.627	0.5759965	0.01265302
2004	4.377	182.912	0.3202132	0.01219936
2005	4.517	185.144	0.3961989	0.01175996
2006	4.696	187.321	0.6069871	0.01133391
2007	4.982	189.444	0.5094195	0.01092045
2008	5.235	191.513	-0.0125812	0.01051870
2009	5.229	193.528	0.7528226	0.01012809
2010	5.622	195.488	0.3909212	0.00974781
2011	5.842	197.393	0.1917983	0.00937737
2012	5.954	199.244	0.3013600	0.00901607
2013	6.134	201.041	0.0104167	0.00866347
2014	6.140	202.782	-0.3847603	0.00831899
2015	5.904	204.469		

GDP and Population are in billions and millions, respectively. Annual growth rates are in %. GDP data was taken from [17].

The method assumes the behavior of GDP by 2060 will follow that of the calibration period, from 2000 to 2015. In this period, the growth rate of the GDP is modeled by a Normal distribution with mean and variance calculated from the data sample. The forecast of the GDP starts from the last observed point and assumes that its growth follows the laws of a Brownian motion [15] up to the forecast horizon, taking as growth rates of the GDP those obtained from the calibration period. So, if *Y*_0_ is the last observed value of the GDP, *a* is the mean of the growth rate of the GDP in the calibration period and *b*^2^ its variance, for a level of confidence *α*, and a time *t* after the last observation of the GDP, the confidence interval is:

$$\begin{array}{c} \left[{\text{Y}}_{0}{\text{e}}^{\text{at + b}\sqrt{\text{t}}\phi^{-1}\left(\frac{1-\alpha }{2}\right)},{\text{Y}}_{0}{\text{e}}^{\text{at + b}\sqrt{\text{t}}\phi^{-1}\left(\frac{1+\alpha }{2}\right)}\right] \end{array}$$ (12)

where *ϕ*^−1^(.) is the inverse of the cumulative distribution function of the standard normal distribution.

An observation on the use of the data, whose sample is rather small: they reflect a recent period of the Brazilian economy for which we have assumed the growth rate of the variable to be stationary. The alternative option, i.e., to use the quarterly GDP values provided by IBGE, would only add seasonal effects without substantially changing our results. The use of time-series statistical techniques, such as autoregressive moving average (ARMA) or autoregressive integrated moving average (ARIMA) [16] for example, could improve short-term forecasts since past oscillations tend to repeat themselves and short-term decisions do not “divert” the process trajectory. In the long run, however, the results are similar.

**Fig 8:**
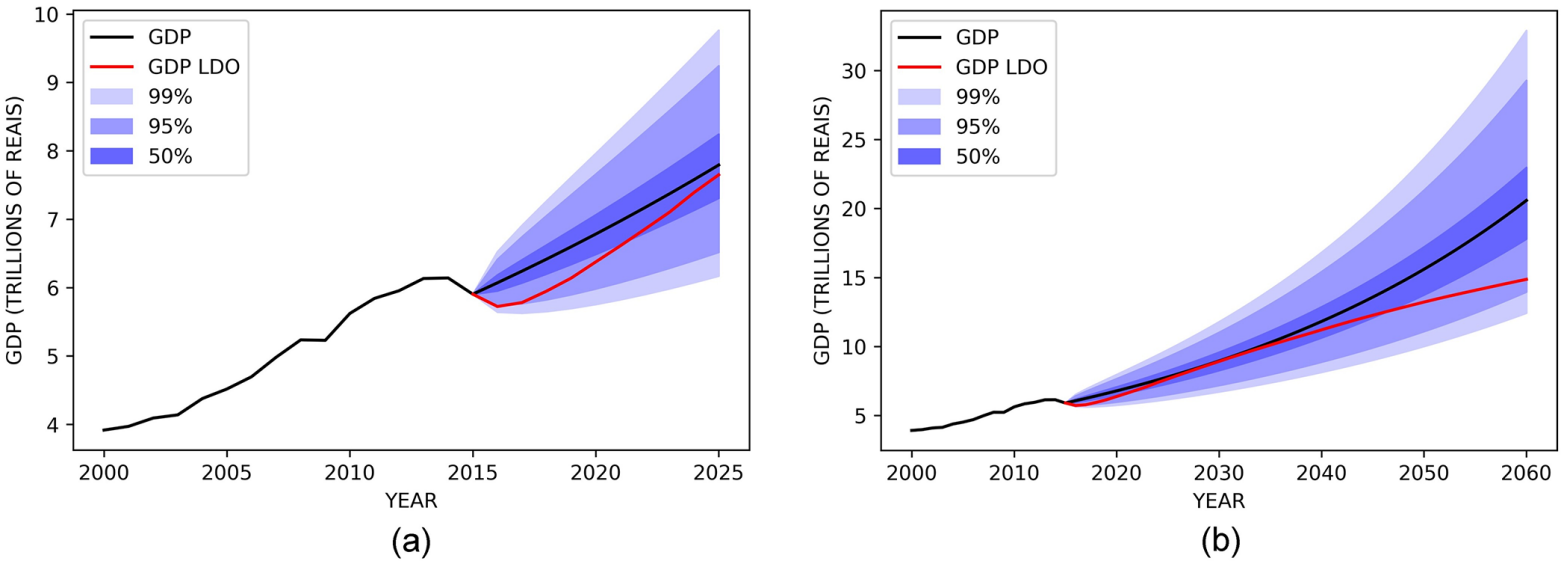
Evolution of the Brazilian GDP between 2000 and 2015 and forecasts until 2025(a) and 2060(b) with confidence intervals of 50, 95 and 99%. The black line from the year 2016 are results using the mathematical modeling approach and the red line are the official numbers from 2017 LDO.

Fig 8 shows Brazilian GDP values from 2000 to 2015 and their projections to 2025 (a) and 2060 (b) using our model. We have added confidence intervals levels of 95%, and 99% and, in the red colored lines, the GDP values as calculated in the official documents. In this case, the 2017 LDO.

The high volatility of the GDP makes its forecast challenging in the short term and practically impossible, with acceptable certainty, in the long run. As seen in both results, 95% and 99% confidence levels generate intervals with a very high margin of error. For comparison purposes, the interval of 50% was drawn to show that only in this case the error margins are at acceptable levels, but with the confidence of a coin toss.

### RGPS Forecasts with dispersion indicators

In the same manner, we can extend our exercise to Social Security actuarial results. Table 7 shows the value of Brazilian GDP and the realized RGPS revenue, expenditure and deficit for the period from 2002 to 2015. At the end of Table 7, we present the mean, standard deviation, and correlation with GDP data. The high correlations of revenue and expenditure with GDP motivated the use of the same method used previously for GDP forecasts. However, it cannot be applied to the RGPS deficit for theoretical reasons that reflect the weak correlation between these variables. In this work, as a first approximation, we have chosen—as explained before—to model the relevant variables (revenue, expenditure, and deficit) directly as a Brownian motion [15].

**Table 7:** GDP, RGPS revenue, expenditure and deficit in millions of reais (2015 prices).

Year	GDP	Revenue	Expenditure	Deficit
2002	4,137,037.67	211,416.44	255,955.00	44,538.56
2003	4,184,234.20	210,891.01	273,269.07	62,378.07
2004	4,425,244.61	228,582.31	302,706.66	74,124.36
2005	4,566,946.73	243,973.40	328,247.70	84,274.30
2006	4,747,888.65	262,109.79	350,592.41	88,482.62
2007	5,036,079.35	284,710.92	370,834.37	86,123.44
2008	5,292,627.07	306,351.89	371,060.48	64,708.59
2009	5,285,968.31	318,166.18	391,793.00	73,626.82
2010	5,683,907.94	341,564.24	409,060.02	67,495.77
2011	5,909,810.48	366,503.20	417,717.99	51,214.79
2012	6,023,348.34	381,411.42	436,508.73	55,097.31
2013	6,204,339.28	395,658.46	458,503.41	62,844.95
2014	6,235,606.40	403,571.86	470,623.25	67,051.39
2015	6,000,570.46	351,467.00	440,079.70	88,612.70
Mean	5,266,686.39	307,598.44	376,925.13	69,326.69
Standard deviation	758,827.47	68,254.62	67,886.75	14,008.49
Correlation with GDP	1	0.9937	0.9839	-0.0733

The proposed forecast exercise consists of extrapolating the series of RGPS revenues, expenditures and deficits—as observed in the years 2000 to 2015—to the target period, assuming that they will strongly influence the future behavior of these variables in the next decades.

**Fig 9:**
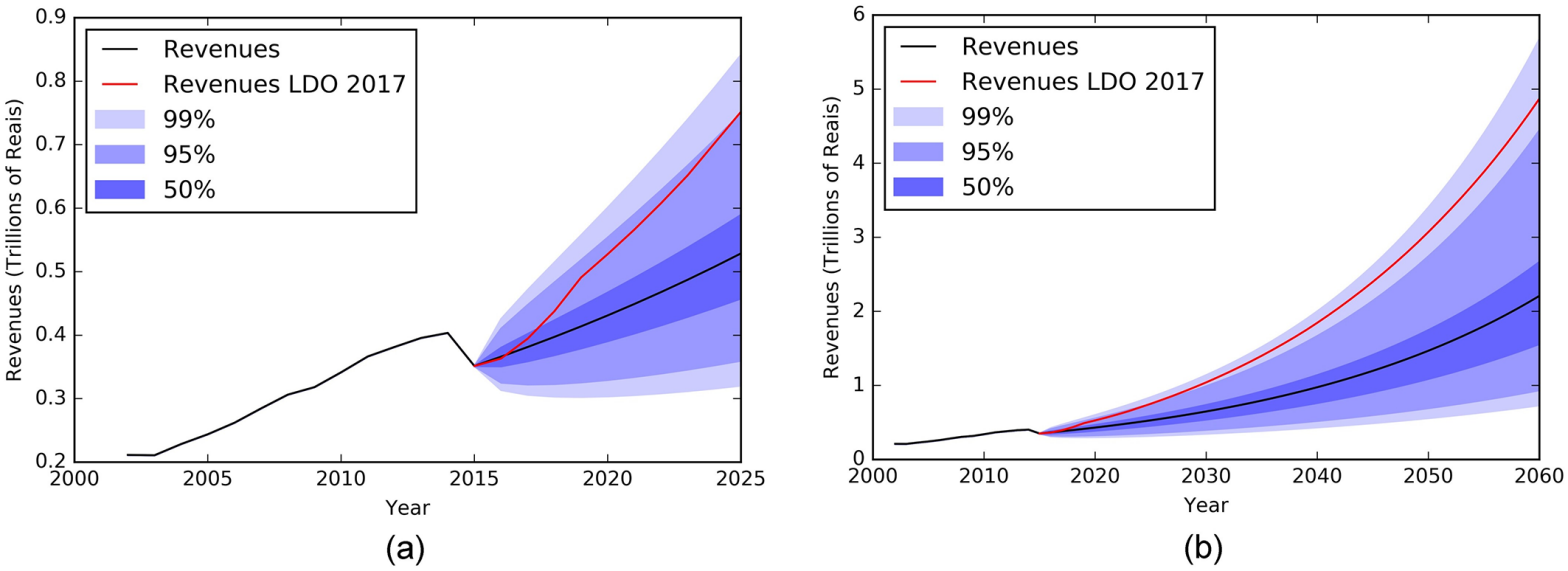
Evolution of RGPS revenue between 2000 and 2015 and forecasts until 2025(a) and 2060(b) with confidence intervals of 50, 95 and 99%. The black line from year 2016 is results using the mathematical modeling approach and the red line is the results from 2017 LDO.

**Fig 10:**
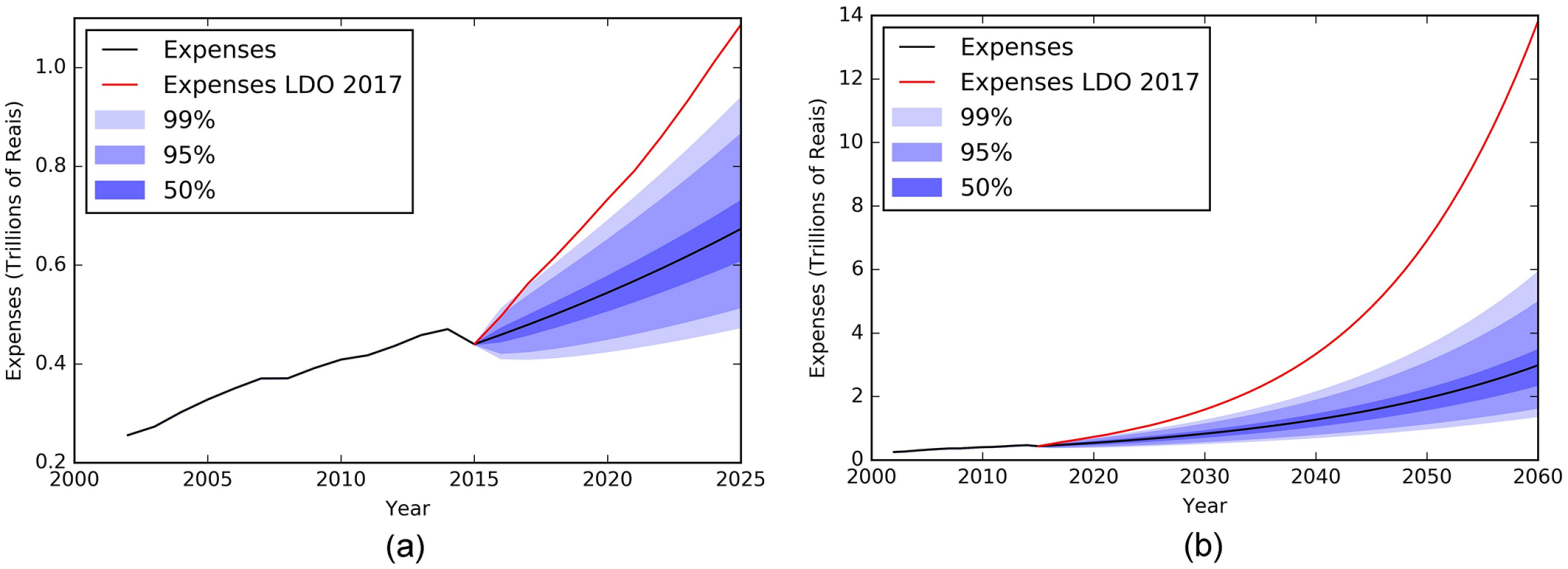
Evolution of RGPS expense between 2000 and 2015 and forecasts until 2025(a) and 2060(b) with confidence intervals of 50, 95 and 99%. The black line from year 2016 is results using the mathematical modeling approach and the red line is the results from 2017 LDO.

**Fig 11:**
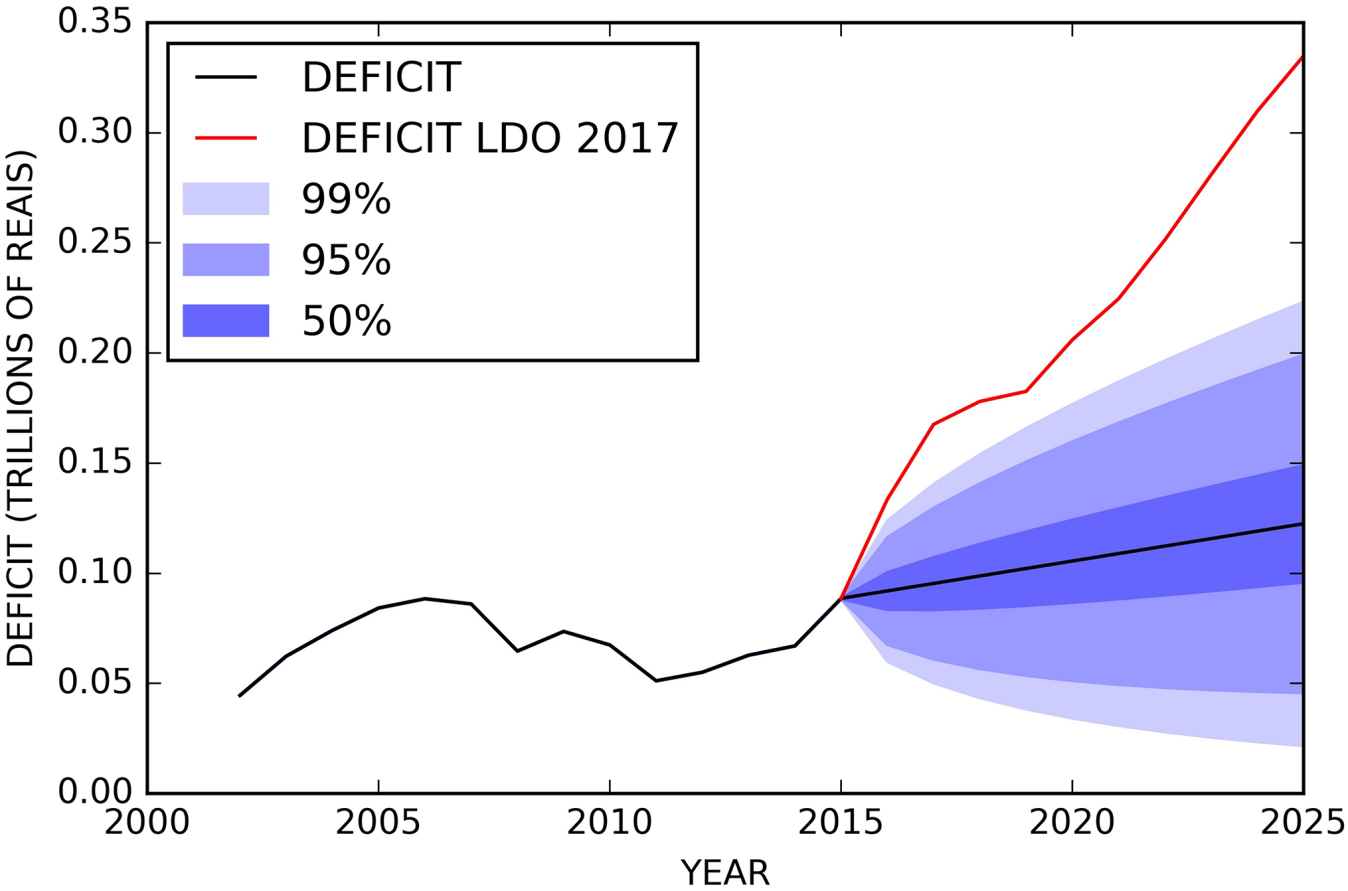
Evolution of RGPS deficit between 2000 and 2015 and forecasts until 2025 with confidence intervals of 50, 95 and 99%. The black line from the year 2016 is results using the mathematical modeling approach and the red line is the results from 2017 LDO.

Figs 9–11 show an extrapolation exercise for the year 2025 and 2060. The blue color gradient represents the confidence interval of 50%, 95%, and 99%. The red curve shows the government forecasts for the same variables in the same periods (values taken from the 2017 LDO).

All data and developed software used to implement this mathematical modeling are available on [18].

From our exercise, we have observed that the government overestimates revenues and especially expenses, which causes the deficit to grow exponentially. As such results represent a structural break in the historical behavior of the studied variables, they naturally raise relevant questions: what aspects provided by the government are influencing so drastically the pension result beyond the aging population problem? What macroeconomic assumptions have been made, directly affecting revenues and expenditures? What is the world scenario and its possible implications for the national economy?

These questions find no answer in the official documents. We insist that the unpredictable behavior of economic variables exposes the fragility of forecasts and that any forecast potentially suffers from this type of shortcoming. Therefore, it is crucial that all assumptions, scenarios and equations involved in the forecasting exercise be made available [13]. More than fabricating certainty, acknowledging uncertainty may be more useful for decision makers in a context of a Social Security reform.

## Conclusions

This paper attempts to make an analysis of the Social Security forecasts presented annually in the budget guidelines of the Brazilian government. Although these forecasts have been reported since 2002, no systematic and comprehensive evaluation of their accuracy has ever been published. We show that the forecasts depicted in the LDO lack reliability in the long term. A self-assessment of the methods used should be performed (and be published) giving a clearer understanding of the limitations of the model.

The failure in our replication attempt of the LDO results demonstrates the lack of transparency in official documents, both in the described equations and in the databases purportedly used. The government should follow best practices in academia and make their forecasting procedures public and replicable, making the model and data used fully available or the presented errors are unlikely to be corrected. Moreover, the unjustified delay in updating data sets used for projections greatly compromises forecast results, impairing the establishment of a robust basis for fine-tuned decision-making in Social Security policy.

We have shown that long-term forecasts of variables such as GDP and Social Security solvency carry a large component of inconstancy and uncertainty that needs to be measured through the definition of confidence intervals. As macroeconomic literature points out, both GDP and Social Security financing are affected by the international environment, the evolution of the productive structure, the macro policy options of each elected government, and by political-institutional issues. The burden of unpredictable factors make any conclusions about forecasts quite vulnerable.

The collective efforts of the scientific community could easily be directed to help the Brazilian government in the challenging task of forecasting Social Security financial series, taking advantage of many of the dramatic improvements in statistical modeling over the last years. More transparent forecasting procedures would also enable members of Congress and partisans on both sides of the alley to consider alternative assumptions explicitly when they debate proposals to ensure the solvency of Social Security.

In our future work, we are developing studies of a Brazilian Social Security forecast model with explicit calibration as done in [19], which we consider representing an improvement in relation to current forecasting practices. We are also developing a free and open source software that allows public simulation and analysis of the financial and social impact of possible pension reforms in Brazil.
